# Contralateral neurofluid dynamics predict survival in IDH wild-type glioblastoma: A DTI-ALPS and free water imaging study

**DOI:** 10.1093/neuonc/noaf242

**Published:** 2025-10-11

**Authors:** Akifumi Hagiwara, Wataru Uchida, Takuya Ozawa, Kaito Takabayashi, Rui Zou, Benjamin M Ellingson, Christina Andica, Junko Kikuta, Toshiaki Akashi, Akihiko Wada, Kanako Kunishima Kumamaru, Koji Kamagata, Osamu Akiyama, Akihide Kondo, Shigeki Aoki

**Affiliations:** Department of Radiology, Juntendo University Graduate School of Medicine, Tokyo, Japan; Department of Radiology, The ­University of Tokyo, Tokyo, Japan; Department of Radiology, Juntendo University Graduate School of Medicine, Tokyo, Japan; Faculty of Health Data Science, Juntendo University, Chiba, Japan; Department of Radiology, Juntendo University Graduate School of Medicine, Tokyo, Japan; Department of Radiology, Juntendo University Graduate School of Medicine, Tokyo, Japan; Department of Radiology, Juntendo University Graduate School of Medicine, Tokyo, Japan; UCLA Brain Tumor Imaging Laboratory, Department of Radiological Sciences, David Geffen School of Medicine, ­University of California Los Angeles, Los Angeles, CA, USA; Department of Radiology, Juntendo University Graduate School of Medicine, Tokyo, Japan; Faculty of Health Data Science, Juntendo University, Chiba, Japan; Department of Radiology, Juntendo University Graduate School of Medicine, Tokyo, Japan; Department of Radiology, Juntendo University Graduate School of Medicine, Tokyo, Japan; Department of Radiology, Juntendo University Graduate School of Medicine, Tokyo, Japan; Department of Radiology, Juntendo University Graduate School of Medicine, Tokyo, Japan; Faculty of Health Data Science, Juntendo University, Chiba, Japan; Department of Radiology, Juntendo University Graduate School of Medicine, Tokyo, Japan; Department of Neurosurgery, Juntendo University School of Medicine, Tokyo, Japan; Department of Neurosurgery, Juntendo University School of Medicine, Tokyo, Japan; Department of Radiology, Juntendo University Graduate School of Medicine, Tokyo, Japan; Faculty of Health Data Science, Juntendo University, Chiba, Japan

**Keywords:** DTI-ALPS, free water imaging, glioblastoma, glymphatic system, neurofluid

## Abstract

**Background:**

Glioblastoma (GBM) may disrupt glymphatic function and neurofluid dynamics locally and in distant brain regions. However, the prognostic relevance of such alterations remains unclear. We investigated whether diffusion tensor image analysis along the perivascular space (DTI-ALPS) and free water (FW) imaging serve as biomarkers of glymphatic dysfunction and survival in patients with IDH wild-type GBM.

**Methods:**

We retrospectively analyzed preoperative MRI data from 277 patients in the UPENN-GBM and 269 patients in the UCSF-PDGM cohorts. The ALPS index was quantified in tumor regions and normal-appearing white matter (NAWM) in both hemispheres, and the FW volume fraction in the contralateral NAWM. Data harmonization was performed using ComBat to adjust for intersite variability. Survival analyses were conducted using log-rank tests and Cox regression models. Optimal ALPS index and FW thresholds were derived from the UPENN-GBM dataset and validated in the UCSF-PDGM.

**Results:**

The ALPS index was significantly lower in tumor regions than NAWM (*P* < .01). In the contralateral hemisphere of the UPENN-GBM cohort, a lower ALPS index and higher FW in NAWM were independently associated with shorter overall survival (HR = 0.75, *P* = .027 for ALPS index; HR = 1.34, *P* = .04 for FW). The identified thresholds successfully stratified survival in UPENN-GBM and were validated in UCSF-PDGM (*P* = .011 for ALPS; *P* = .038 for FW).

**Conclusions:**

Neurofluid dynamic alterations in the contralateral hemisphere, assessed using DTI-ALPS and FW imaging, were independently associated with survival in patients with IDH wild-type GBM. These findings support the use of glymphatic imaging markers for prognostic stratification and therapeutic targeting.

## Graphical abstract

**Graphical Abstract:**
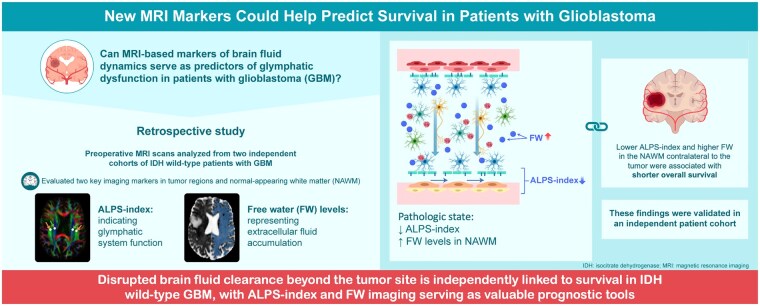

Key Points

- Analysis along the perivascular space (ALPS) index and free water (FW) in the contralateral white matter predict survival in glioblastoma.
- Thresholds from ALPS and FW imaging stratify risk in independent GBM cohorts.
- Regions of interest overlap with tumor affects ALPS index interpretation in GBM.

Importance of the Study

This study demonstrates that alterations in neurofluid dynamics in brain regions distant from the primary tumor, as measured using novel MRI techniques (DTI-ALPS and FW imaging), are associated with survival in patients with IDH wild-type glioblastoma, independent of tumor volume. These findings suggest that glymphatic system dysfunction may represent a systemic phenomenon influencing patient outcomes. The identified imaging biomarkers could potentially enhance risk stratification and inform treatment planning—for instance, by directing high-risk patients (low ALPS/high FW) toward intensified or immunomodulatory regimens—and may also serve as longitudinal readouts for therapies targeting neuroinflammation or fluid balance.

Glioblastoma (GBM), an isocitrate dehydrogenase (IDH) wild-type, grade 4 glioma according to the WHO classification of central nervous system (CNS) tumors, remains one of the most aggressive primary brain tumors, with a dismal prognosis. Despite advances in standard multimodal treatment—including maximal safe surgical resection, radiation therapy, and chemotherapy with temozolomide—the median survival time for patients with GBM remains approximately 11–15 months, and the 5-year survival rate is less than 5%.^1^ These poor outcomes underscore the urgent need for novel prognostic biomarkers and therapeutic targets based on a deeper understanding of GBM pathophysiology beyond the conventional focus on tumor cells alone.

Recent evidence suggests that the glymphatic system may play a role in the pathophysiology of brain tumors. The glymphatic system is a brain-wide perivascular pathway that mediates the exchange of cerebrospinal fluid (CSF) and interstitial fluid (ISF), facilitating the clearance of soluble proteins, metabolites, and waste products from the CNS.^2^ This system comprises a network of perivascular spaces surrounding cerebral blood vessels, through which CSF enters the brain parenchyma, mixes with ISF, and removes waste via perivenous drainage pathways. Its function is facilitated by aquaporin-4 (AQP4) water channels expressed on astrocytic endfeet that ensheath the cerebral vasculature.^3^ Accumulating evidence indicates that dysfunction of the glymphatic system, which tends to decline with age, contributes to various neurological disorders, including Alzheimer’s disease, traumatic brain injury, and stroke, by impairing the clearance of neurotoxic waste.^4^^,^^5^ Recent studies suggest that gliomas can impair glymphatic function not only in the tumor-bearing hemisphere but also in the contralateral hemisphere. Mechanistically, tumor-induced vasogenic edema and elevated intracranial pressure may compress the perivascular space, disrupting CSF–ISF exchange.^6^ Additionally, remote neuroinflammation extending to the contralateral hemisphere—as observed in GBM^7^^,^^8^—can impair astrocytic AQP4 polarization and promote interstitial edema, further hindering glymphatic clearance.^9^^,^^10^ Moreover, glymphatic dysfunction may impair immune surveillance by limiting antigen drainage to the cervical lymph nodes, potentially promoting tumor progression.^10^^,^^11^

Several techniques have been developed to indirectly assess neurofluid dynamics in vivo using MRI.^12^ One such method, diffusion tensor image analysis along the perivascular space (DTI-ALPS), was recently introduced to evaluate water diffusion along perivascular spaces, providing an indirect measure of glymphatic system function.^13^ The ALPS index, derived from this technique, quantifies the ratio of water diffusion along the perivascular space to that in the perpendicular direction, with lower values potentially indicating reduced perivascular flow. Additionally, free water (FW) imaging—based on a two-compartment model that separates extracellular FW from cellular water diffusion—has emerged as a promising approach to quantify changes in extracellular fluid volume.^14^ An increased FW volume fraction in the white matter has been associated with interstitial fluid stasis and neuroinflammation in various neurological conditions.^15^^,^^16^ The ALPS index has been applied to study neurofluid dynamics in neurodegenerative and neuroinflammatory diseases, including Alzheimer’s disease and multiple sclerosis.^15^^,^^17^ Recent studies have extended the application of the ALPS index to brain tumors, particularly gliomas, revealing consistent evidence of glymphatic dysfunction associated with tumor characteristics.^18–22^ Lower ALPS index values have been reported in high-grade gliomas compared to low-grade gliomas and in IDH wild-type tumors compared to IDH-mutant tumors.^18-20^^,^^22^ Furthermore, increased tumor and edema volumes have shown significant negative correlations with ALPS index values.^19^^,^^21^^,^^22^ Notably, bilateral ALPS index assessment has demonstrated that contralateral glymphatic dysfunction is associated with shorter overall survival (OS), highlighting the clinical relevance of glymphatic imaging in glioma prognosis.^21^ However, in IDH wild-type GBM—the most aggressive subtype—the prognostic significance of ALPS index alterations remains unclear. Moreover, prior studies have not distinguished between tumor-infiltrated tissue and unaffected regions within the ipsilateral hemisphere.^18^^,^^19^ These limitations underscore the need for more granular investigations into glymphatic impairment in GBM.

Therefore, in this study, we aimed to evaluate the utility of DTI-ALPS and FW imaging as potential biomarkers for assessing neurofluid dynamics in patients with IDH wild-type GBM (**Figure 1**). Specifically, we sought to (1) compare the ALPS index between tumor regions and normal-appearing white matter (NAWM) in both ipsilateral and contralateral hemispheres, and (2) investigate the relationship between the ALPS index, FW volume fraction, and patient survival across two independent datasets, and determine an optimal threshold for distinguishing patients with better versus worse survival outcomes. By elucidating the relationship between neurofluid dynamics and GBM prognosis, the ALPS index may serve as a novel imaging biomarker for risk stratification and treatment planning—for example, by identifying high-risk patients (low ALPS/high FW) who may benefit from intensified or immunomodulatory therapies. It may also serve as a longitudinal readout for treatments targeting neuroinflammation or fluid balance, and inform the development of therapeutic strategies aimed at restoring glymphatic function.

**Figure 1.**
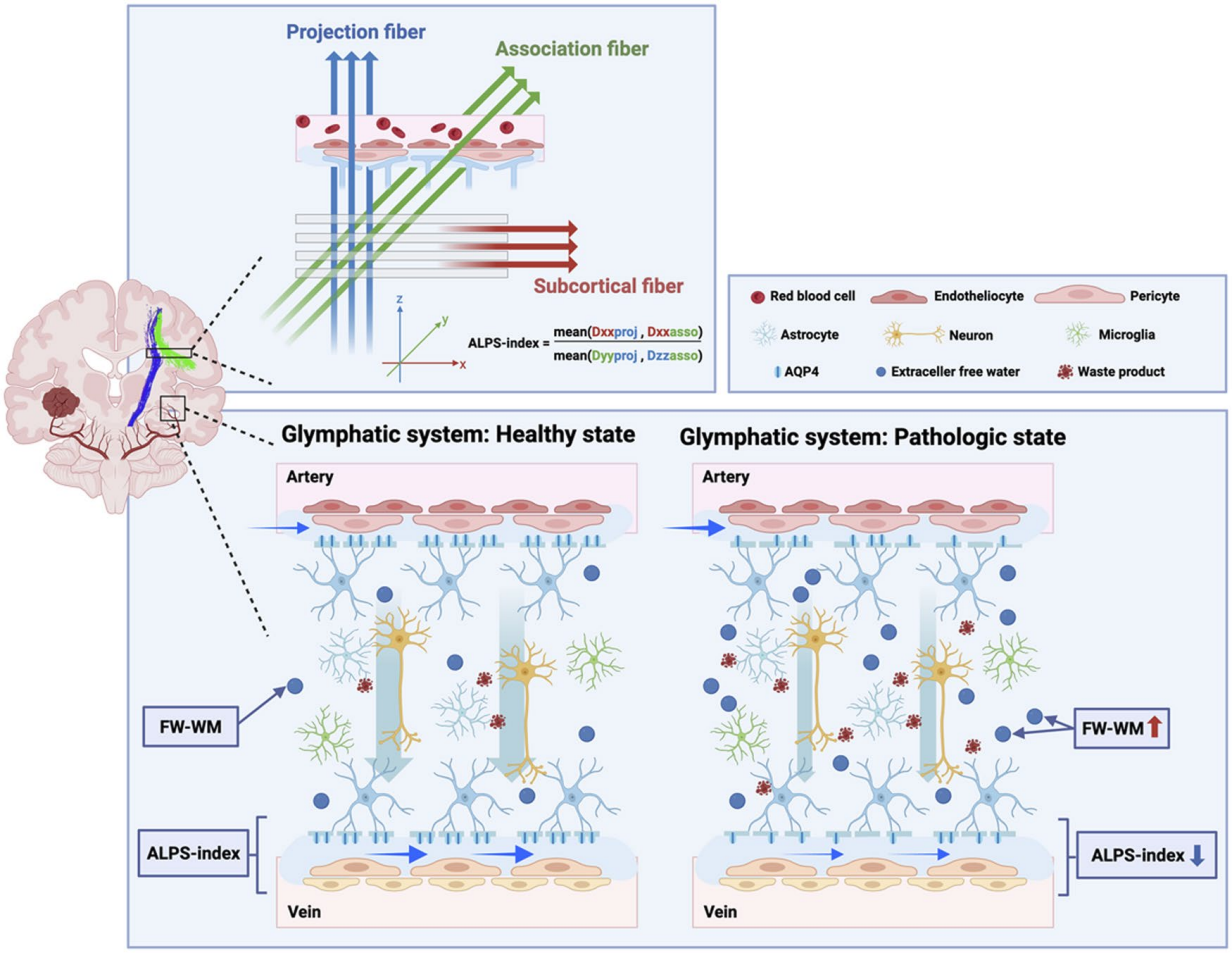
Schematic representation of diffusion tensor image analysis along the perivascular space (DTI-ALPS) computation and glymphatic system alterations in healthy and pathologic states. (Top left): Conceptual overview of the ALPS index derived from DTI. Fractional anisotropy (FA) maps highlight projection (blue), association (green), and subcortical (red) fibers. The perivenous cerebrospinal fluid (CSF) outflow pathway runs orthogonal to both projection and association fiber tracts. Consequently, diffusivity along the x-axis, which is perpendicular to these dominant fiber orientations, serves as a surrogate marker for interstitial fluid movement along perivascular spaces and thereby enables the indirect assessment of glymphatic transport efficiency. (Bottom panels): Schematic illustration of the glymphatic system in the (left) healthy state and the (right) pathologic state, such as in brain regions contralateral to gliomas. In the healthy condition, CSF flows through periarterial spaces, enters the brain parenchyma via aquaporin-4 (AQP4) channels on astrocyte endfeet, mixes with interstitial fluid (ISF), and is subsequently cleared via perivenous pathways. This functional fluid exchange is associated with a higher ALPS index and lower extracellular free water in white matter (FW-WM). In contrast, under pathologic conditions—even in areas remote from the tumor—glymphatic dysfunction may arise, leading to impaired AQP4 polarity, inefficient waste clearance, and interstitial fluid accumulation. These changes are reflected by a reduced ALPS index and elevated FW-WM, indicating compromised glymphatic activity. Created in BioRender. Hagiwara, A. (2025) https://BioRender.com/85x2eyi

## Materials and Methods

### Study Participants and Demographics

**Figure 2.**
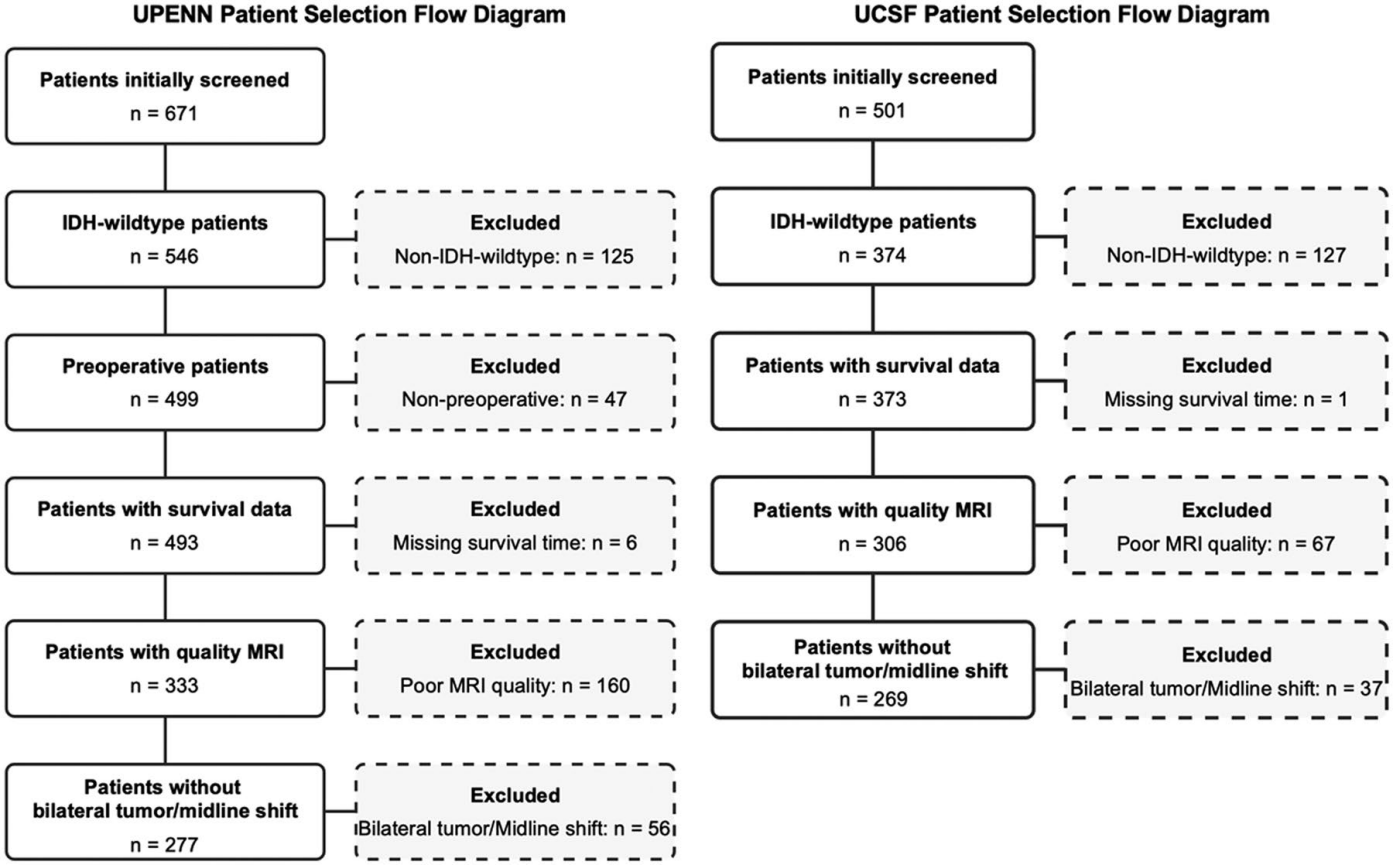
Flow chart of participant selection.

This retrospective study utilized data from 2 publicly available cohorts: UPENN-GBM^23^ and UCSF-PDGM.^24^ Patients were included if they had histologically confirmed IDH-wildtype glioblastoma and available preoperative MRI and survival data. Individuals were excluded if they had poor-quality MRI scans, presented with bilateral tumors, or exhibited a midline shift greater than 5 mm, in order to minimize confounding effects on contralateral hemisphere measurements. The complete selection process is illustrated in **Figure 2**. All data were obtained from de-identified, open-access repositories (TCIA), with IRB approval and informed consent secured by the original data providers. No additional ethical approval was required for this secondary analysis.

### Image Acquisition

Pretreatment MRI scans were used to obtain clinical imaging data for each patient included in this study. Detailed descriptions of the acquisition protocols are available in the original publications of the UPENN-GBM^23^ and UCSF-PDGM^24^ datasets and on the TCIA website (https://www.cancerimagingarchive.net/). Notably, diffusion-weighted imaging (DWI) in the UPENN-GBM dataset was performed with b-values of 0 and 1000 s/mm^2^ using 31–124 diffusion directions, whereas the UCSF-PDGM dataset used a b-value of 0 and 2000 s/mm^2^ with 56 diffusion directions. These acquisitions enabled full DTI modeling in both datasets.

### Image Preprocessing

All DWI data underwent preprocessing using a pipeline implemented in MRtrix3. This included denoising based on Marchenko-Pastur principal component analysis,^25^ correction for Gibbs ringing artifacts,^26^ eddy current and motion correction,^27^ and bias field correction for low-frequency intensity non-uniformity.^28^ The FMRIB Software Library (FSL; ver 6.0.7) was used to generate DTI maps and compute diffusivity maps in the three axes: x-axis (right-left; $D_{xx}$), y-axis (anterior-posterior; $D_{yy}$), and z-axis (inferior-superior; $D_{zz}$). For tumor region segmentation, we utilized preexisting segmentation labels available in the datasets, which included enhancing tumor, necrotic core, and peritumoral edematous and/or infiltrated non-enhancing tissue. As histopathological confirmation was not available, we acknowledge that these non-enhancing FLAIR hyperintense regions may contain a variable mixture of vasogenic edema and infiltrative tumor. Tumor regions of interest (ROIs) were registered to the preprocessed DWI images using Advanced Normalization Tools (ANTs; version 2.5.4), based on pre-contrast T1-weighted images.

### ALPS Index

For ALPS index calculation, fractional anisotropy (FA) maps from all participants were initially registered to the high-resolution FMRIB58_FA standard-space template using linear registration, followed by nonlinear registration with Advanced Normalization Tools (ANTs; version 2.5.4).^29^ To identify an optimal reference subject for ROI placement, we calculated the sum of squared differences between each participant’s registered FA map and the template. The participant whose FA map exhibited the smallest sum of squared differences relative to the FMRIB58_FA template was selected as the reference subject. On this subject’s color-coded FA map, we placed spherical ROIs (4-mm diameter) bilaterally in the corticospinal tract (projection fibers, visualized as blue areas in the FA map, corresponding to superoinferior direction) and the adjacent white matter (association fibers, visualized as green areas in the FA map, corresponding to anteroposterior direction) adjacent to the lateral ventricular bodies (Figure S1). Using ANTs registration tools, these ROIs were transformed into each participant’s native FA space. Manual corrections were performed to ensure accurate anatomical placement of the ROIs for each participant. After verification, the ALPS index was calculated using the following formula:


$$\mathrm{ALPS}-\mathrm{index}=\frac{\mathrm{mean}(D_{xx,\;\mathrm{proj}},\;D_{xx,\;\mathrm{assoc}})}{\mathrm{mean}(D_{yy,\;\mathrm{proj}},\;D_{zz,\;\mathrm{assoc}})}$$

Here, $D_{xx,\;\mathrm{proj}}$ denotes the diffusivity along the x-axis ($D_{xx}$) measured in the projection fiber region; $D_{xx,\;\mathrm{assoc}}$ represents the diffusivity along the x-axis obtained from the association fiber region; $D_{yy,\;\mathrm{proj}}$ is the diffusivity along the y-axis ($D_{yy}$) in the projection fiber region, and $D_{zz,\;\mathrm{assoc}}$is the diffusivity along the z-axis ($D_{zz}$), as measured in the association fiber region. A lower ALPS index theoretically reflects decreased water diffusion along the perivascular space.^13^ The ALPS indices calculated with *b*-values of 1000 and 2000 s/mm were shown to be comparable in a study by Okazawa et al,^30^ and diffusivity measured across different field strengths is theoretically consistent.^31^

Furthermore, based on overlap between the ROIs and the tumor, the ROIs were categorized as (1) contralateral normal-appearing white matter (cNAWM), (2) ipsilateral normal-appearing white matter (iNAWM), (3) FLAIR hyperintense tumor, or (4) contrast-enhancing tumor (Figure S1).

### FW Imaging

For FW imaging analysis, we generated cNAWM masks from 3D T1-weighted images using FSL's FAST segmentation algorithm.^32^ This mask was subsequently co-registered to diffusion-weighted images using ANTs.^29^ To calculate the mean FW volume fraction in cNAWM, we employed a regularized bi-tensor model.^14^ Unlike previous approaches, our method utilized an advanced initialization technique described by Parker et al,^33^ which improves the accuracy and reliability of FW estimation by optimizing the initial parameters for the bi-tensor fitting procedure. A higher FW in the white matter is generally recognized as reflecting interstitial fluid stasis,^14^^,^^15^ which in turn reflects reduced glymphatic function in the brain.^34^ However, it is well established that FW estimation is most accurate when using two or more b-values, whereas single b-value acquisitions are considered suboptimal for this purpose.^35^^,^^36^ Nevertheless, to explore the clinical utility of FW as a prognostic biomarker and to maximize the value of routinely acquired clinical DWI data, we proceeded with FW estimation despite this limitation.

### Harmonization

To mitigate intersite variability, we applied ComBat harmonization^37^^,^^38^ to both the ALPS index and FW values across datasets. This batch effect correction was performed prior to statistical analyses to minimize the influence of acquisition-related differences on these metrics. A previous study showed that the ComBat algorithm effectively reduced site-specific variance in diffusion MRI while preserving biologically meaningful signals,^39^ supporting its use in harmonizing the ALPS index and FW in this multi-cohort study. To preserve the variance attributable to individual characteristics, age and sex were included as covariates in the combat model.

### Statistical Analysis

In the UPENN-GBM dataset, a one-way analysis of variance (ANOVA), followed by Tukey’s post hoc test, was used to compare ALPS index values between groups categorized by ROI. The ALPS indices measured in the iNAWM and cNAWM and FW values measured in the cNAWM were dichotomized at the median into high and low groups for survival analysis. Survival was assessed using the log-rank test, as well as univariate and multivariate Cox regression analyses, with age, sex, and enhancing tumor volume included as covariates for OS. Because only a small subset of cases had ROIs overlapping with tumor tissue, ALPS indices from these regions were excluded from survival analysis. Additionally, to assess the influence of white matter integrity on ALPS index measurements, we included mean fractional anisotropy (FA) and mean diffusivity (MD) values from the association and projection fiber ROIs as covariates. To address potential site-specific effects, separate analyses were performed for the UPENN and UCSF cohorts.

For groups showing statistical significance in survival analysis in the UPENN-GBM dataset, we determined the optimal threshold—defined as the point with the lowest p-value and highest hazard ratio (HR)—by performing log-rank tests at incremental points across the metric range. In the UCSF-PDGM dataset, the cNAWM and iNAWM groups were dichotomized using the optimal ALPS index and FW thresholds into high and low groups, respectively, and log-rank tests were conducted. Furthermore, to evaluate the combined prognostic utility of the ALPS index and FW, we performed an additional stratified survival analysis by dividing patients into three groups based on their ALPS and FW status relative to the identified optimal thresholds: (1) high ALPS/low FW, (2) high ALPS/high FW or low ALPS/low FW, and (3) low ALPS/high FW. Log-rank tests were used to compare OS across these groups.

A significance level of 5% was considered statistically significant in all analyses. Statistical analyses were performed using GraphPad Prism (version 10.4; GraphPad Software) or MATLAB (version R2023b; MathWorks, Inc.).

## Results

### Patient Characteristics

Demographic and clinical characteristics of the patients included in the analysis are summarized in **Table 1**. Patients were stratified based on whether the ALPS index ROIs were placed in NAWM or overlapped with FLAIR hyperintense tumors, contrast-enhancing tumors, or necrosis. Across both UPENN-GBM and UCSF-PDGM datasets, the mean age ranged from 61.3 to 65.4 years, depending on the ROI category. A small number of cases were excluded from the group comparisons because the ALPS index ROIs overlapped with areas of tumor necrosis.

**Table 1.** Patient characteristics stratified by ROI location relative to tumor

UPENN-GBM/UCSF-PDGM	Contralateral hemisphere	Ipsilateral hemisphere			Exclusion from analysis for ipsilateral hemisphere
	cNAWM	iNAWM	FLAIR hyperintense tumor	Contrast-enhancing tumor	Tumor necrosis
**Number of subjects**	277/269	188/167	66/75	16/24	7/3
**Age (mean ± SD)**	63.8 ± 11.4/62.2 ± 11.8	63.4 ± 10.9/61.3 ± 12.1	65.4 ± 11.9/63.4 ± 10.9	63.7 ± 11.3/63.7 ± 11.7	-
**Sex (Male: Female)**	169:108/155:114	124:64/101:66	36:30/40:35	6:10/12:12	-
**Contrast-enhancing tumor volume [ml]**	15.0 ± 12.4/19.2 ± 14.0	14.3 ± 12.6/15.7 ± 12.2	16.6 ± 12.6/22.0 ± 13.9	14.9 ± 9.0/32.8 ± 15.8	-

Abbreviations: cNAWM, contralateral normal-appearing white matter; iNAWM, ipsilateral normal-appearing white matter.

### Comparison of ALPS Indices Across Different Brain Regions

**Figure 3.**
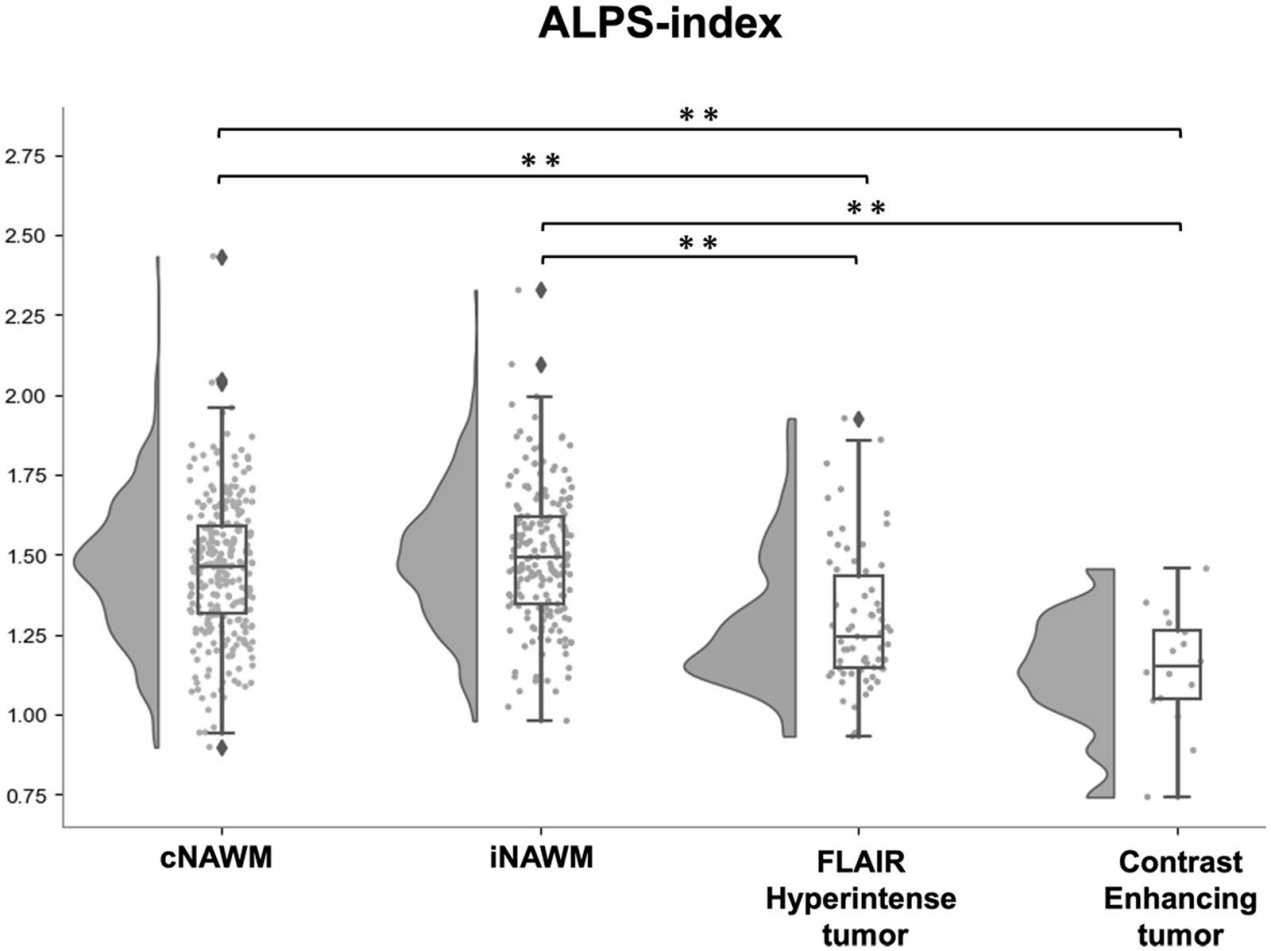
Comparison of the ALPS index between normal-appearing white matter (NAWM) in the ipsilateral hemisphere, FLAIR hyperintense tumor, contrast-enhancing tumor, and NAWM in the contralateral hemisphere (**P < .01).

Compared with the NAWM in both contralateral and ipsilateral hemispheres, the ALPS index was significantly lower in FLAIR hyperintense tumors and contrast-enhancing tumors (all *P*s < .01) (**Figure 3**). No significant differences were observed between the cNAWM and iNAWM.

### Survival Analysis Based on the ALPS Index and FW

Cox regression analysis demonstrated that the ALPS index and FW in cNAWM were significantly associated with survival time in both univariate and multivariate analyses adjusted for age, sex, and tumor volume, except for FW in cNAWM of the UCSF-PDGM cohort in the multivariate analysis (**Table 2**; multivariate analysis: *P* = .027, HR = 0.75, 95% CI 0.59–0.97 for the ALPS index in the UPENN-GBM cohort; *P* = .04, HR = 1.34, 95% CI 1.01–1.76 for FW in the UPENN-GBM cohort; *P* = .02, HR = 0.67, 95% CI 0.48–0.93 for the ALPS index in the UCSF-PDGM cohort; and *P* = .20, HR = 1.24, 95% CI 0.89–1.74 for FW in the UCSF-PDGM cohort). Furthermore, the ALPS index in the cNAWM was significantly associated with OS, even after additionally controlling for FA and MD in the ROIs of the association and projection fibers as surrogates of local white-matter integrity (**Table 2**; *P* = .049, HR = 0.78, 95% CI 0.60–1.00 in the UPENN-GBM cohort; and *P* = .029, HR = 0.69, 95% CI 0.49–0.96 in the UCSF-PDGM cohort). The ALPS index in iNAWM showed no significant association with survival time.

**Table 2.** Univariate and Multivariate Cox Regression Analysis of overall survival (OS)

	OS (Univariate)			OS (Multivariate)^a^			OS (Multivariate)^b^		
	*P* value	Z score	HR (95%CI)	*P* value	*Z* score	HR (95%CI)	*P* value	*Z* score	HR (95%CI)
**UPENN-GBM**									
ALPS in the cNAWM	.002*	−3.16	0.68 (0.53–0.86)	.027*	−2.21	0.75 (0.59–0.97)	.049*	−1.97	0.78 (0.60–1.00)
ALPS in the iNAWM	.20	−1.27	0.83 (0.61–1.11)	.419	−0.81	0.89 (0.61–1.11)	.433	−0.78	0.88 (0.64–1.21)
FW in the cNAWM	<.001***	3.40	1.51 (1.19–1.92)	.04*	2.06	1.34 (1.01–1.76)	NA	NA	NA
Age	<.001***	4.20	1.02 (1.01–1.04)	NA	NA	NA	NA	NA	NA
Sex	.80	−0.26	0.97 (0.76–1.24)	NA	NA	NA	NA	NA	NA
enhancing tumor volume	.007**	2.68	1.39 (1.09–1.77)	NA	NA	NA	NA	NA	NA
**UCSF-PDGM**									
ALPS in the cNAWM	.003**	−3.00	0.61 (0.44–0.84)	.02*	−2.42	0.67 (0.48–0.93)	.029*	−2.19	0.69 (0.49–0.96)
ALPS in the iNAWM	.35	−0.94	0.82 (0.55–1.24)	.64	−0.47	0.90 (0.59–1.38)	.97	−0.03	0.99 (0.64–1.55)
FW in the cNAWM	.017*	2.39	1.47 (1.07–2.02)	.20	1.28	1.24 (0.89–1.74)	NA	NA	NA
Age	<.001***	4.43	1.04 (1.02–1.05)	NA	NA	NA	NA	NA	NA
Sex	.38	0.88	1.15 (0.84–1.59)	NA	NA	NA	NA	NA	NA
enhancing tumor volume	.29	0.86	1.15 (0.84–1.57)	NA	NA	NA	NA	NA	NA

a Controlling for age, sex, and enhancing tumor volume.

b Controlling for age, sex, enhancing tumor volume, and FA and MD in the association and projection fibers.

* *P* < .05,

** *P* < .01, and.

*** *P* < .001.

Abbreviations: cNAWM, contralateral normal-appearing white matter; iNAWM, ipsilateral normal-appearing white matter.

In the contralateral hemisphere, the high-ALPS group and low-FW group showed significantly longer OS than the low-ALPS and high-FW groups, respectively, in the UPENN-GBM cohort (*P* = .001, HR = 0.68, 95% CI 0.54–0.87, median OS 17.5 vs 12.7 months for the ALPS index; *P* < .001, HR = 0.66, 95% CI 0.52–0.85, median OS 17.2 vs 11.6 months for FW) (Figure S2). In the ipsilateral hemisphere, no significant difference in OS was observed between high- and low-ALPS groups.

**Figure 4.**
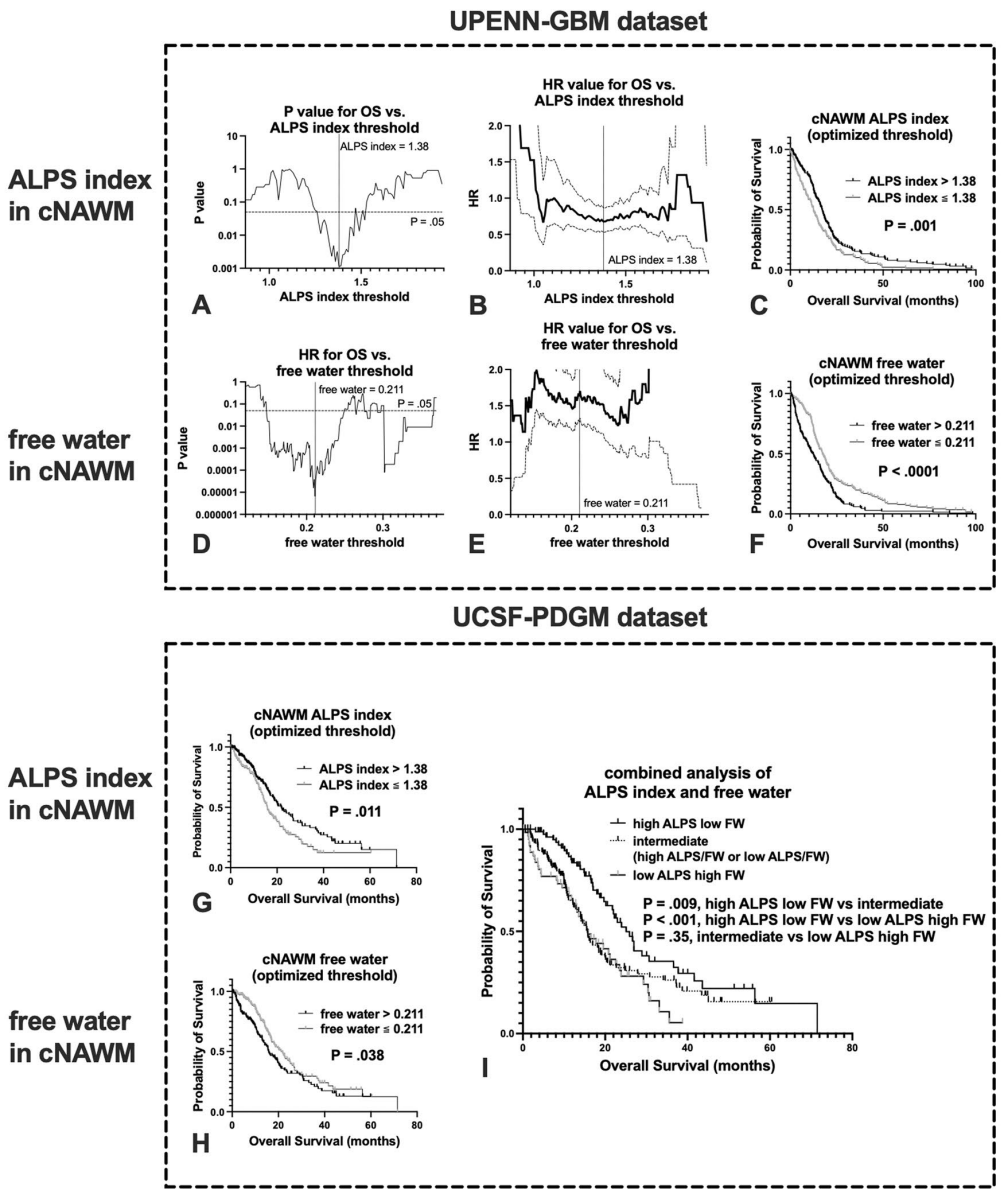
(A, D) *P* values from log-rank tests assessing overall survival (OS) across varying thresholds of the ALPS index and free water (FW) in the normal-appearing white matter (NAWM) contralateral to the tumor in the UPENN-GBM dataset. The lowest *P* values (.011 for the ALPS index and <.001 for FW) were observed at thresholds of 1.38 and 0.211, respectively. (B, E) Hazard ratios (HR, solid black line) and 95% CIs (dashed lines) for OS based on different ALPS index and FW thresholds in GBM patients. (C, F) Kaplan-Meier plots showing significantly longer OS in patients with the ALPS index > 1.38 and FW ≤ 0.211, respectively, in the UPENN-GBM cohort. (G, H) Optimized thresholds identified from the UPENN-GBM dataset significantly stratified OS in the independent UCSF-PDGM cohort. (I) Kaplan-Meier plot showing overall survival stratified into three groups based on combined ALPS index and FW thresholds in the UCSF-PDGM cohort: (1) high ALPS/low FW, (2) discordant profiles (high ALPS/high FW or low ALPS/low FW), and (3) low ALPS/high FW. Patients with high ALPS and low FW demonstrated significantly longer survival compared to the other groups.

When we looped through the ALPS index in cNAWM in increments of 0.01 in the UPENN-GBM cohort, thresholding with ALPS indices of 1.27–1.52 resulted in significant results, with variable numbers of patients in groups with better and worse survival. The lowest *P* value of .011 was achieved at an ALPS index of 1.38, grouping 126 patients with worse OS and 151 patients with better OS (**Figure 4**). When we looped through the FW in cNAWM in increments of 0.001, thresholding with an FW value of 0.148–0.366 resulted in significant results, with variable numbers of patients in groups with better and worse survival. The lowest *P*-value of <.001 was achieved at an FW of 0.211, grouping 117 patients with worse OS and 160 patients with better OS (**Figure 4**). When these optimal cutoff values were applied to the UCSF-PDGM dataset, OS was also significantly longer with higher ALPS index and lower FW in cNAWM (*P* = .011, HR = 0.67, 95% CI 0.49–0.93, median OS 21.8 vs 15.8 months for the ALPS index; *P* = .038, HR = 0.72, 95% CI 0.52–0.99, median OS 21.8 vs 15.7 months for FW, **Figure 4**). Similarly, when patients in the UCSF-PDGM cohort were stratified into three groups based on the combination of ALPS index and FW thresholds, OS was significantly longer in the high ALPS/low FW group (83 patients) compared to the intermediate group (121 patients) (*P* = .009, HR = 0.61, 95% CI 0.42–0.88, median OS 25.1 vs 15.7 months, **Figure 4**) and the low ALPS/high FW group (65 patients) (*P* < .001, HR = 0.48, 95% CI 0.31–0.77, median OS 25.1 vs 15.9 months), while no significant difference was observed between the intermediate and low ALPS/high FW groups (*P* = .35, HR = 0.83, 95% CI 0.56–1.24, median OS 15.7 vs 15.9 months).

## Discussion

In this study, we demonstrated that the ALPS index was significantly lower in tumor-involved regions, including FLAIR hyperintense and contrast-enhancing areas, than in NAWM in both hemispheres. Furthermore, a higher ALPS index and lower FW in the NAWM contralateral to the tumor were significantly associated with longer OS in patients with IDH wild-type GBM. These associations remained significant even after adjusting for enhancing tumor volume and, for the ALPS index, local white-matter microstructure (FA and MD), except for the FW values in the UCSF-PDGM dataset, indicating that these markers capture a distinct pathophysiological dimension that is not reflected by conventional imaging biomarkers. Although no statistically significant differences were observed between ALPS index values in iNAWM and cNAWM, only the cNAWM values were significantly associated with overall survival. One possible explanation is that the iNAWM, despite appearing normal on conventional MRI, may harbor subclinical tumor infiltration or be affected by peritumoral microenvironmental changes such as subtle edema, altered extracellular matrix, or disrupted white matter tracts.^40^ These alterations may introduce measurement noise or reduce the prognostic specificity of ALPS and FW metrics in the ipsilateral hemisphere. Our finding that iNAWM and cNAWM demonstrated comparable mean ALPS values yet differed in their prognostic associations highlights an unresolved biological question. At present, it is not known to what extent microscopic infiltration or edema in iNAWM diminishes the prognostic specificity of ALPS and FW metrics, or how subtle pathological involvement of cNAWM is sufficient to drive the observed survival associations. Importantly, the presence of abnormal ALPS and FW values in brain regions remote from the tumor may reflect a more global disturbance of neurofluid dynamics or systemic disease burden, which could explain why these contralateral measures show stronger associations with outcome. Addressing these issues will likely require studies that combine advanced imaging with histopathological validation and spatially resolved molecular analyses. Such work could help establish the biological and quantitative thresholds at which these imaging biomarkers remain informative for prognosis.

The ALPS index quantifies water diffusivity along perivascular spaces and has been proposed as a non-invasive surrogate of glymphatic function.^13^ Recent studies have shown that lower ALPS index values track with delayed intrathecal contrast clearance in cerebral small-vessel disease^41^ and with greater amyloid burden in early Alzheimer’s disease,^42^ underscoring its pathophysiological relevance. However, emerging evidence indicates that the ALPS index may also be influenced by various pathological processes, including local neurodegeneration.^43^^,^^44^ The significantly lower ALPS index observed in tumor regions compared to the NAWM implies that the ALPS index may be affected by tumor infiltration. Notably, previous studies evaluating the ALPS index in the tumor hemisphere did not report whether the ROIs overlapped with tumor tissue.^18^^,^^19^ To the best of our knowledge, the present study is the first to explicitly compare ALPS index values between ROIs overlapping with tumor tissue and those placed outside the tumor in the ipsilateral hemisphere. Therefore, when assessing the ALPS index in the hemisphere ipsilateral to the tumor, specific types of ROIs should be clearly defined and reported to ensure appropriate interpretation.

The observed association between a reduced ALPS index, an elevated FW, and shorter OS in our study may reflect globally impaired glymphatic function in cNAWM. This dysfunction is potentially mediated by neuroinflammation, vasogenic edema, and elevated intracranial pressure.^7^^,^^21^ A recent study demonstrated that the contralateral ALPS index in patients with glioblastoma was lower than that in healthy controls,^20^ reinforcing the pathological nature of this alteration. Importantly, the prognostic value of the ALPS index and FW in the cNAWM remained significant even after adjusting for enhancing tumor volume. Furthermore, the ALPS index maintained a significant association with OS even after additional adjustment for local white-matter microstructure metrics such as FA and MD. Although FA and MD are themselves influenced by perivascular space anatomy and glymphatic function,^45^ their inclusion as covariates aimed to partially control for the confounding effects of white matter microstructural damage. The persistence of these associations suggests that ALPS and FW may capture distinct pathophysiological factors beyond tumor burden and local white-matter integrity. Furthermore, when patients were stratified into three groups based on combined ALPS index and FW thresholds, those with both high ALPS and low FW demonstrated significantly longer survival compared to other groups. Notably, patients with discordant marker profiles (high ALPS/high FW or low ALPS/low FW) showed survival outcomes similar to those with combined adverse profiles (low ALPS/high FW), indicating that the presence of an unfavorable marker in either ALPS or FW is associated with worse prognosis. These findings suggest that glymphatic dysfunction (low ALPS) and fluid accumulation (high FW) may have additive or complementary effects on patient outcomes, and that even subtle impairments in neurofluid dynamics, captured by either marker, can negatively influence survival. However, given that GBM is known to exhibit diffuse infiltration throughout the brain, including the contralateral hemisphere and even the spinal cord, it is conceivable that abnormal ALPS and FW values in the contralateral NAWM may reflect microscopic tumor spread undetectable by standard anatomical MRI. This potential confounder should be considered when interpreting these imaging markers as indicators of global glymphatic impairment. Although both ALPS index and FW are considered surrogate markers of glymphatic function, they likely reflect distinct and partially overlapping aspects of neurofluid dynamics. The ALPS index is influenced not only by water diffusivity along perivascular spaces but also by local microstructural alterations in white matter,^43^ while FW primarily quantifies extracellular FW but has also been associated with neuroinflammatory processes.^16^ Therefore, each measure may be subject to non-glymphatic confounders; yet, together, these measures provide complementary insights into glymphatic dysfunction. Emerging evidence indicates that impaired glymphatic clearance may exacerbate neuroinflammation through multiple mechanisms, including the accumulation of inflammatory cytokines, disruption of aquaporin-4 polarization, and perivascular infiltration of immune cells.^9^^,^^10^ Glymphatic impairment may facilitate tumor progression by sustaining a pro-inflammatory microenvironment, impeding waste clearance, or attenuating immune surveillance by limiting antigen drainage to the cervical lymph nodes.^46^^,^^47^ However, although these mechanisms are biologically plausible, causality cannot be established from our data, and further studies are required.

Importantly, our results were replicated in an independent dataset (UCSF-PDGM), where threshold-based survival stratification using the ALPS index and FW yielded a similar prognostic separation. This strengthens the generalizability of our findings and supports the robustness of DTI-derived neurofluid markers across sites and scanner protocols, particularly after ComBat harmonization. Although single b-value DWI is suboptimal for FW estimation,^35^^,^^36^ our findings suggest that even clinically acquired DWI sequences can provide prognostically meaningful information if properly modeled and harmonized. Moreover, given that both the UPENN-GBM and UCSF-PDGM datasets are publicly available, readers may reproduce our analysis by applying similar harmonization techniques and utilizing the optimal ALPS index and FW thresholds identified in this study to perform prognostic evaluations in patients with glioblastoma.

The findings of this study have several clinical implications. Imaging-derived markers of glymphatic function may aid in early risk stratification, particularly in patients with similar tumor burdens. Additionally, the ALPS index and FW could potentially serve as pharmacodynamic biomarkers for monitoring the response to therapies targeting edema, inflammation, or glymphatic flow, such as bevacizumab, AQP4 modulators, or immunotherapy.^48^^,^^49^ In the era of precision neuro-oncology, incorporating neurofluid biomarkers into multiparametric MRI protocols may provide a system-level perspective that complements existing anatomical and molecular imaging techniques.

Nonetheless, this study has some limitations. Our analysis was retrospective and was dependent on publicly available datasets with inherent heterogeneity. Although we applied rigorous pre-processing and harmonization steps, variations in image acquisition and ROI registration may have introduced bias. The estimation of FW volume fraction was performed using single-shell DTI. Single-shell acquisitions are known to limit the precision of bi-tensor modeling,^35^^,^^36^ and this should be considered when interpreting the FW results. It is important to note that both the ALPS index and FW volume fraction are surrogate markers of glymphatic function. The exact biological underpinnings of these imaging-derived metrics are not fully established, and caution is warranted when interpreting them as direct measures of glymphatic activity. Further experimental and clinical validation is required to better define their mechanistic relevance. Furthermore, although we controlled for several confounders, residual bias related to treatment heterogeneity, steroid use, and systemic inflammation could not be excluded. Additionally, we note that the FLAIR hyperintense regions classified as tumor may represent edema and/or infiltrative non-enhancing tumor tissue, as conventional MRI cannot reliably distinguish between the two without histological validation. This ambiguity may influence the interpretation of diffusion-based measurements in such regions. Finally, the ALPS ROI was situated adjacent to the lateral ventricle within the subventricular zone, a region known to be susceptible to GBM cell infiltration.^50^ Consequently, even on the contralateral side of the primary lesion, the possibility of tumor infiltration cannot be entirely excluded.

## Conclusions

Our findings highlight the prognostic significance of neurofluid dynamics, particularly in the hemisphere contralateral to the tumor, in patients with IDH wild-type glioblastoma. DTI-ALPS and FW imaging offer promising noninvasive tools for assessing glymphatic dysfunction and interstitial fluid status. Future studies should focus on prospective validation, integration with molecular and immunological profiling, and exploration of these biomarkers as therapeutic response indicators.

## Supplementary Material

noaf242_Supplementary_Data

## Data Availability

All data used in this study are publicly available and were obtained from two open-access datasets. The UPENN-GBM dataset can be accessed via The Cancer Imaging Archive (TCIA) at https://www.cancerimagingarchive.net/collections/. No proprietary or patient-identifiable data were used. All analyses were conducted on de-identified data and no additional restrictions were applied to data access.
