# Corrigendum to: Contralateral neurofluid dynamics predict survival in IDH wild-type glioblastoma: A DTI-ALPS and free water imaging study

**DOI:** 10.1093/neuonc/noag056

**Published:** 2026-04-10

## Abstract

**Graphical Abstract:**
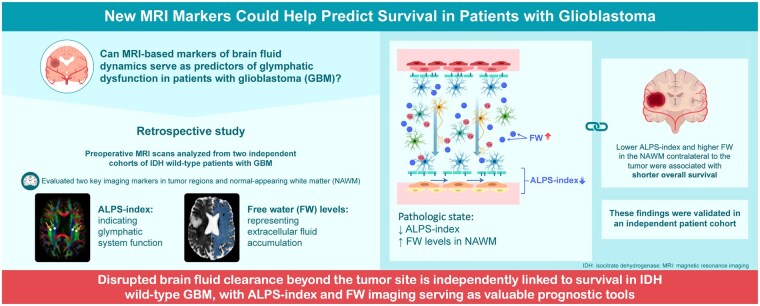

This is a corrigendum to: Akifumi Hagiwara, Wataru Uchida, Takuya Ozawa, Kaito Takabayashi, Rui Zou, Benjamin M Ellingson, Christina Andica, Junko Kikuta, Toshiaki Akashi, Akihiko Wada, Kanako Kunishima Kumamaru, Koji Kamagata, Osamu Akiyama, Akihide Kondo, Shigeki Aoki, Contralateral neurofluid dynamics predict survival in IDH wild-type glioblastoma: A DTI-ALPS and free water imaging study, *Neuro-Oncology*, Volume 28, Issue 1, January 2026, Pages 299–310, https://doi.org/10.1093/neuonc/noaf242

In the originally published version of the paper the graphical abstract was missing. This has now been supplied to the paper.

