# Supplementary material for: Contralateral neurofluid dynamics predict survival in IDH wild-type glioblastoma: A DTI-ALPS and free water imaging study
Source: Neuro Oncol. 2025 Oct 11;28(1):299–310. doi: 10.1093/neuonc/noaf242 (PMC12962648; doi:10.1093/neuonc/noaf242)
Supplement: noaf242_Supplementary_Data [file noaf242_supplementary_data.docx]

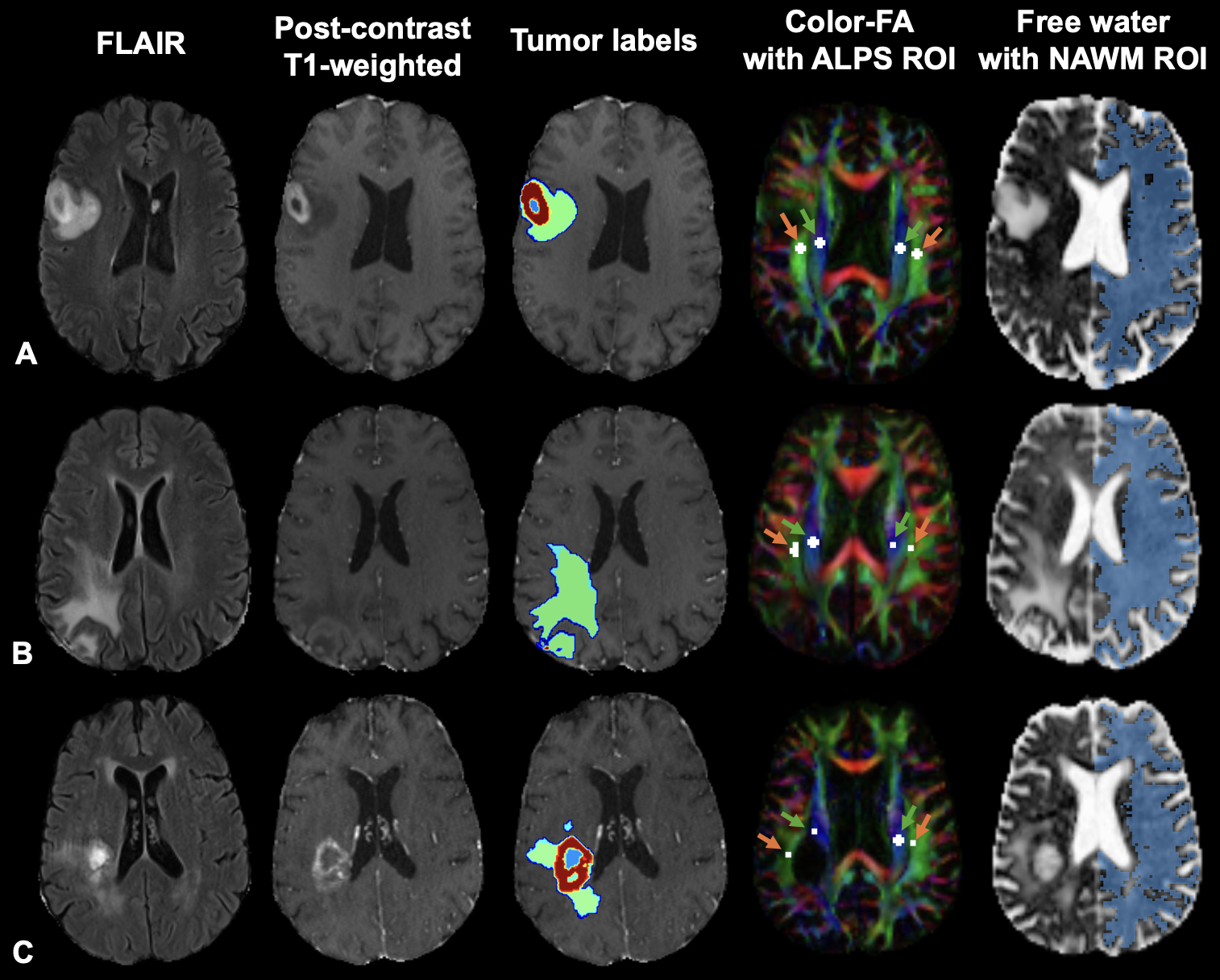


**Supplementary Fig. 1.** Representative cases illustrating ALPS region of interest (ROI) placement relative to tumor regions in patients with isocitrate dehydrogenase (IDH) wild-type glioblastoma. Each row (A–C) shows FLAIR, post-contrast T1-weighted images, tumor labels, color-FA maps with ALPS ROIs, and free water (FW) maps with normal-appearing white matter (NAWM) ROIs contralateral to the tumor. In (A), ALPS ROIs in the tumor hemisphere do not overlap with the tumor. In (B), the ROIs overlap with FLAIR hyperintense tumor, and in (C), they overlap with the contrast-enhancing tumor. Tumor labels are overlaid on the post-contrast T1-weighted images and indicate FLAIR hyperintense tumor (light green), contrast-enhancing tumor (red), and necrosis (blue). On the color-FA maps, orange and green arrows indicate association and projection fiber ROIs, respectively. On the FW maps, ROIs in the contralateral NAWM are shown in light blue.


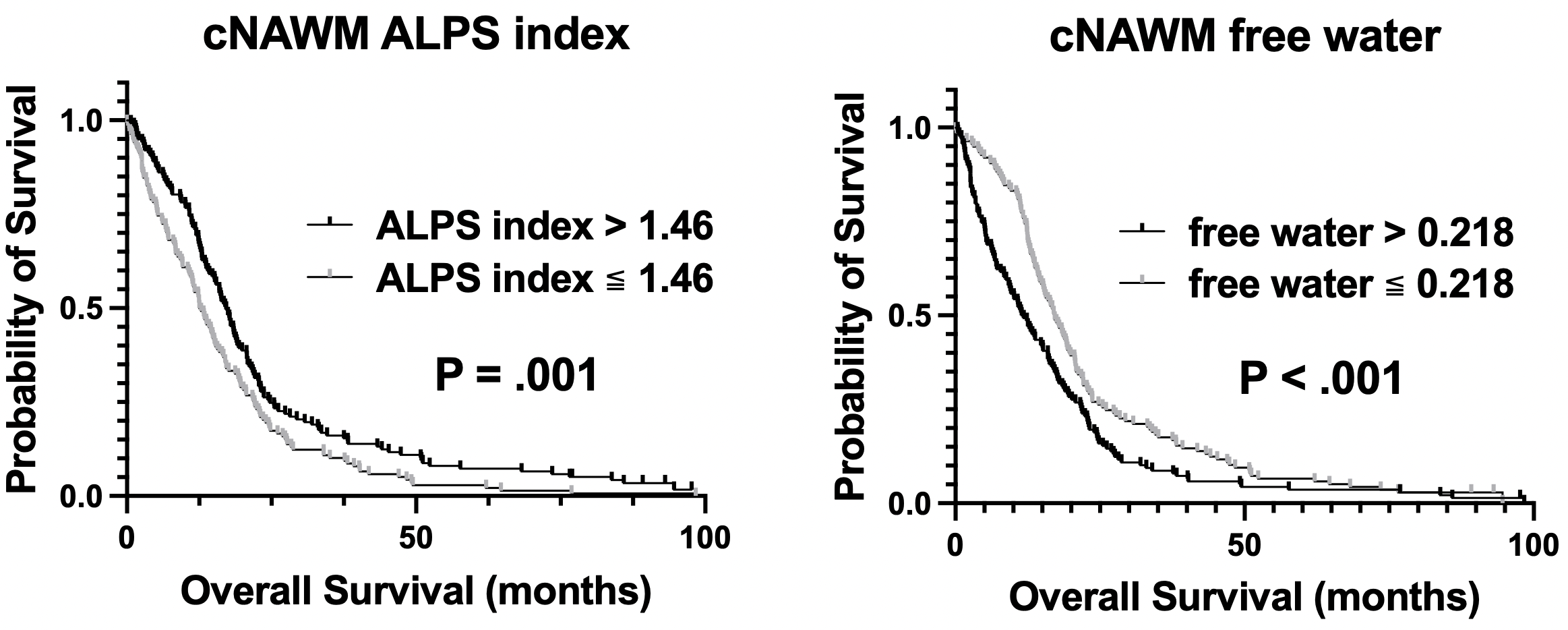


**Supplementary Fig. 2.** Kaplan–Meier plots comparing overall survival (OS) between groups stratified by the median values of ALPS index and free water (FW) in the normal-appearing white matter (NAWM) of the hemisphere contralateral to the tumor in the UPENN-GBM cohort. Patients with an ALPS-index > 1.46 and FW ≤ 0.218 showed significantly longer OS.
